# A guide into the world of high-resolution 3D imaging: the case of soft X-ray tomography for the life sciences

**DOI:** 10.1042/BST20210886

**Published:** 2022-03-08

**Authors:** Chidinma Adanna Okolo

**Affiliations:** Beamline B24, Diamond Light Source, Harwell Science and Innovation Campus, Didcot, Oxfordshire OX11 0DE, U.K.

## Abstract

In the world of bioimaging, every choice made determines the quality and content of the data collected. The choice of imaging techniques for a study could showcase or dampen expected outcomes. Synchrotron radiation is indispensable for biomedical research, driven by the need to see into biological materials and capture intricate biochemical and biophysical details at controlled environments. The same need drives correlative approaches that enable the capture of heterologous but complementary information when studying any one single target subject. Recently, the applicability of one such synchrotron technique in bioimaging, soft X-ray tomography (SXT), facilitates exploratory and basic research and is actively progressing towards filling medical and industrial needs for the rapid screening of biomaterials, reagents and processes of immediate medical significance. Soft X-ray tomography at cryogenic temperatures (cryoSXT) fills the imaging resolution gap between fluorescence microscopy (in the hundreds of nanometers but relatively accessible) and electron microscopy (few nanometers but requires extensive effort and can be difficult to access). CryoSXT currently is accessible, fully documented, can deliver 3D imaging to 25 nm resolution in a high throughput fashion, does not require laborious sample preparation procedures and can be correlated with other imaging techniques. Here, we present the current state of SXT and outline its place within the bioimaging world alongside a guided matrix that aids decision making with regards to the applicability of any given imaging technique to a particular project. Case studies where cryoSXT has facilitated a better understanding of biological processes are highlighted and future directions are discussed.

## Introduction

Recent developments in biological imaging have unlocked new potential in data capture and are promising to deliver exciting and impactful insights across an array of biological specimens [1–4]. However, in the world of microscopy, there is usually a trade-off associated with ‘deliverables’ in any optical setup and no one system can satisfy all user requirements in their entirety [5,6]. For instance, techniques such as electron microscopy (EM) deliver data to nanometer resolution but trade-off many other ‘desirables’ leading to: (a) restricted fields of view, (b) time-consuming highly specialized workflows, (c) limited depth of beam penetration requiring sample sectioning or milling and (d) demanding data collection and data processing pipelines [7–12]. The latter impose limitations on the ability of high-end imaging systems to comprehensively image biological samples in a resource-efficient way with regards to personnel hours, overall equipment/facility access and reagent costs. On the other extreme techniques such as confocal microscopy can deliver sub-par resolution (200 nm laterally and 600 nm axially) when compared with EM but are capable of (a) high throughput imaging of dynamic processes, (b) with wider fields of view, (c) optical sectioning and (d) aa requirement for less laborious sample preparation [13,14].

**Figure 1.**
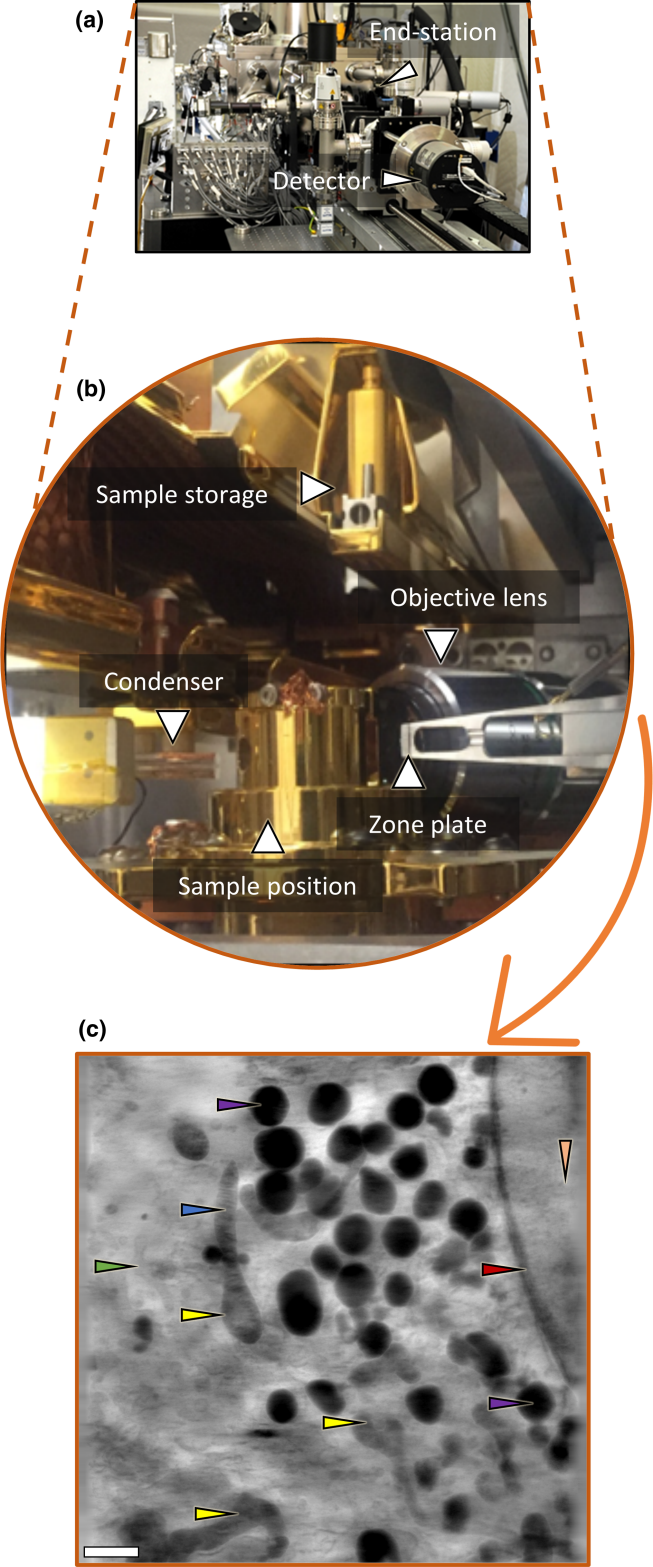
An exemplar full-field transmission X-ray microscope end station at beamline B24, Diamond Light Source. (**a**) A view of the outer components of the microscope's end station. (**b**) Different components of the end station including optics and sample storage. (**c**) Minimum intensity projection of a 3D reconstructed tomogram derived from tilt series captured by the X-ray detector- *Image courtesy* *of Dr Angus Wann (University of Oxford, U.K.)*. Arrows are pointing at different cellular components: green- endoplasmic reticulum, yellow- mitochondria, blue- cristae, red- nuclear envelope, purple- lipid droplets, orange- nucleus. Scale bar = 1 µm.

In the region between nanometer EM resolution and comprehensive 3D imaging to hundreds of nanometers, soft X-ray tomography at cryogenic temperatures (cryoSXT) is a microscopy technique which straddles these capabilities and allays several of the concerns regarding trade-offs in deliverable outcomes in cellular imaging. In this review, soft X-ray tomography (SXT) and cryoSXT will be used interchangeably to mean the same. SXT 3D imaging through native cellular absorption contrast is done using X-ray light in the ‘water window’ spectra area [15–17] where carbon-rich biological structures obstruct light as it passes through them as opposed to the oxygen-rich surrounding medium that does not (Figure 1). As a result, the impression left on a detector when an SXT image is recorded is a negative projection of the cell structure exactly like in the case of medical X-ray imaging (Figure 1c). In fact, in concept and optical implementation, this method is the cellular equivalent of a full-body CT scan in a medical setting, adapted for the microworld of cells. Since soft X-ray imaging depends on cellular content alone, there is no requirement for contrast-enhancing chemicals to be added and therefore the information captured is not altered in any way by foreign material. An additional benefit comes from the fact that, in order to help cells endure sustained exposure to X-ray light during imaging, samples are snap frozen before use, thereby perfectly preserving the exact instant of their lives, without any changes to the structures [18–22]. Hence, dynamic processes can be captured as they unfold through a series of stills snapped at different time points. In implementation, cryoSXT is a breakthrough in optical performance and versatility and is currently driving discoveries far beyond the diffraction limit of conventional light microscopy while permitting the combination of other microscopy techniques [23–25]. Given a soft X-ray source (plasma or synchrotron-based), SXT is delivered through full-field transmission X-ray microscope (TXM) equipped with X-ray optics such as zone plates and capillary condensers. Advances in X-ray optics have meant that imaging to 10 nm optical resolution is possible [26,27], though to provide a practical size of the depth of focus for 3D imaging, 25 nm is currently the best on-the-ground image resolution that is on offer for potential users at selected third-generation synchrotrons to date [28–30,25,31,32].

**Figure 2.**
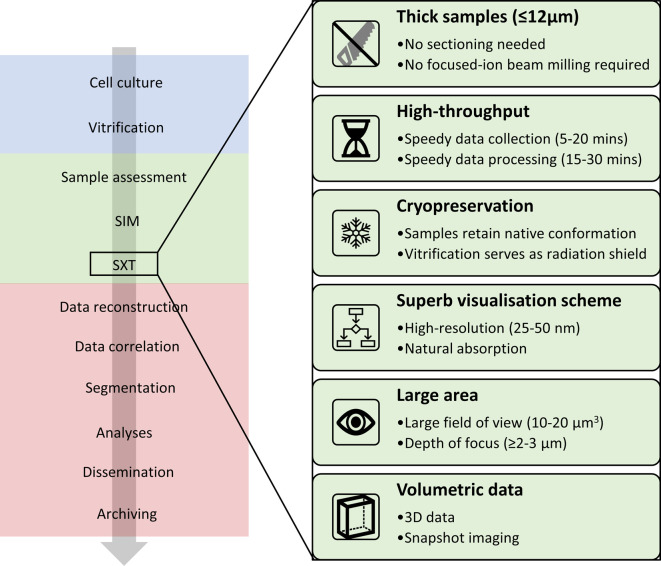
Imaging workflow at the correlative cryo-imaging beamline B24 at the U.K. national synchrotron, Diamond Light Source. This platform takes samples from the preparatory stage through mapping and imaging using fully correlated SXT and super-resolution fluorescence 3D cryo-imaging leading to content-rich data collection, automated processing (provided that there are appropriate fiducial markers) and information harvest.

The requirement for cryogenic sample preparation imposes a degree of difficulty in the adoption of this technique by investigators. However, robust sample preparation workflows for cryoSXT have now been documented [33–35] and these can guide new users to reproducibly deliver high-quality samples for imaging (Figure 2). In brief, where a transmission electron microscopy (TEM) grid is used as a sample carrier, cells are deposited on its surface in solution (used immediately or allowed to adhere and proliferate depending on the requirements of each experiment) and the loaded grid is removed from the medium, gently blotted to remove excess media without drying or flattening the cells and vitrified through rapid immersion in cryogenic liquids [21,22,33,35]. A carrier prepared in this fashion is thereafter ready to be loaded on a TXM for SXT data collection. Advantageously, cryoSXT can image thick samples (up to 10 µm) without loss in resolution (25–40 nm depending on the objectives used), hence, sectioning and milling are not required for cell imaging [22,24,29]. If the imaging subject is tissue, then thin slices of up to 10 μm need to be produced and laid on TEM grids for imaging or alternatively the tissue matter can be dissociated and deposited on grids as a mixed cell population which could then be incubated further to recover cell–cell associations before imaging. Alternative sample presentation involves cell uptake in thin capillaries (single-cell clearance through the opening) which are then cryo-preserved and mounted to a TXM [28].

**Figure 3.**
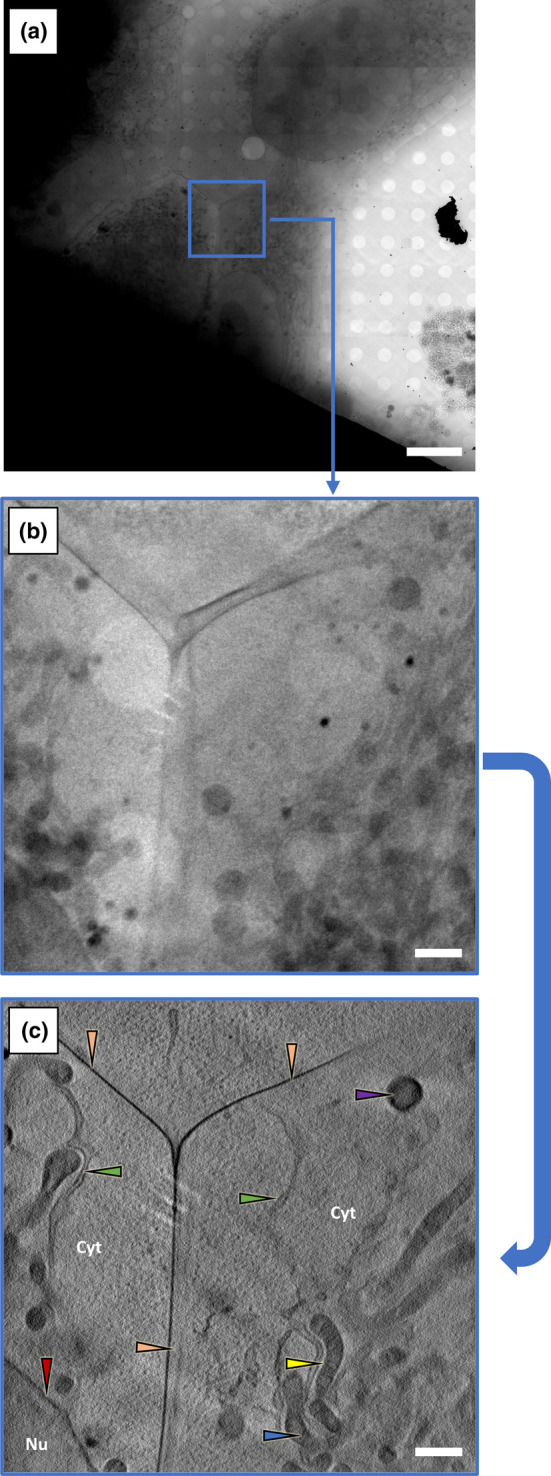
Data collection strategy for SXT. (**a**) A 7 × 7 2D mosaic collected with reference applied and background subtracted. (**b**) A single projection of the area boxed blue in (**a**) during tilt series data acquisition. (**c**) A slice of a reconstructed 3D tomogram of the same area in (**b**), showing a junction between two cells. Scale bars: (**a**) 10 µm (**b**) and (**c**) 1 µm. Arrows are pointing at different cellular components: green- endoplasmic reticulum, yellow- mitochondria, blue- cristae, red- nuclear envelope, purple- lipid droplets, orange- cell membrane. Cyt = cytoplasm, Nu = nucleus. Image courtesy of Dr Nina Vyas (Diamond Light Source, U.K.).

SXT data are collected in a conventional tomography fashion whereupon tilt series are collected (a sum of projections through the sample at different angles to reconstruct 3D information from 2D stills); the field of view of single captures can extend to 20 × 20 µm^2^ and cumulative volumes typically have heights up to 10 μm with a depth of focus of 2–3 μm or more depending on optical performance (beyond which resolution degrades the further we move towards the periphery) [22,30,36–38]. However, by using reconstruction schemes or optimized image acquisition pipelines, artifacts arising from the limited depth of focus can be mitigated [36,38,39]. SXT data collection at a synchrotron setting can be very fast: 5–20 min per tilt series depending on: (a) the exposure time per projection (ranging from 0.5 to 5 s), (b) maximum angles of rotation (−70° to +70° or −90° to +90° depending on the setup used) and (c) angular step increase (0.2–2° steps) [22,29]. With this in mind, a single sample which typically contains a hundred or more of cells, can be used to collect many tilt series per day and therefore SXT is ideal for phenotyping cell populations or teasing statistically significant features with respect to the cellular response to a variety of cues, chemical or environmental (Figure 3). Raw SXT tilt series data are then reconstructed to 3D tomograms using dedicated primarily open-source algorithms such as IMOD [40]. These tomograms are essentially Z-stacks of images that capture the near-physiological ultrastructure in the intra and intercellular environment under examination to high resolution (Figure 3).

In this review, we examine the role of cryoSXT in cellular imaging as a synchrotron radiation-based technique and showcase the instrumentation and implementation underpinning the microscope at B24 at the U.K. national synchrotron facility, Diamond Light Source.

## SXT instrumentation and beamline case study

The broader academic environment has access to synchrotron-based SXT facilities via peer-review processes that allows projects to access resident infrastructure and expertise. There are currently six SXT beamlines worldwide (Table 1) at various stages of development: (1) beamline B24, Diamond Light Source, U.K.; (2) beamline 09 (MISTRAL), ALBA, Spain; (3) beamline 07W SXM beamline, NSRL, China; (4) beamline U41, HBZ, Germany; (5) beamline 2.1, ALS, U.S.A.; (6) beamline 24A, TPS, Taiwan- *under construction* [25,28–32]. Each of these facilities has appropriate support facilities and techniques in addition to water window SXT. Typically, X-ray light is supplied by a bending magnet or insertion device located on the synchrotron ring and is directed via a combination of optics which condition, select the desired energies and focus the light towards the TXM. Once there, X-rays are focused onto the sample by a capillary glass or Fresnel zone plate (FZP) objective lens followed by a FZP objective lens which focuses the formed image to a detector [22,28,30].

**Table 1.** A summary of the SXT beamlines worldwide

Beamline	Source	Energy range, eV	Spatial resolution (nm)	Additional techniques (to water window SXT)	Status
Diamond, U.K. Beamline B24	Bending magnet	200–2600	25–40	CryoSIM Phase-contrast	Operational
ALBA, Spain Beamline Mistral	Bending magnet	270–1200	25–40	XANES CryoSIM	Operational Under construction
NSRL, China SXM Beamline	Bending magnet	200–2500	30	Phase-contrast	Operational
HBZ, Germany Beamline U41	Undulator	180–2800	25–40	NEXAFS*	Operational
ALS, U.S.A. Beamline 2.1	Bending magnet	400–1300	35–80	Cryogenic fluorescence microscope	Operational
TBS, Taiwan Beamline 24A	Bending magnet	260–2600	50	CryoSIM	Under construction

* NEXAFS = near-edge X-ray absorption fine structure.

As an example of the application, we can focus on the correlative cryo-imaging beamline B24 at the U.K. synchrotron Diamond Light Source which is a SXT beamline dedicated to the Life Sciences. Beamline B24 provides semi-automated 3D X-ray absorption-contrast imaging to 25 nm spatial resolution for cryo-preserved biological samples mounted on 3 mm TEM grids coated with perforated carbon support films such as Quantifoil [41]. Alongside X-ray imaging, 3D cryo-fluorescence imaging using SIM is available at the beamline and allows the same sample to be imaged under different contrasting regimes enabling the unambiguous same-sample correlation of 3D volumes captured from the same areas of interest. Data collection, processing and correlation all employ tailor-made software applications that make the capture of useful and high-resolution information expedient and efficient [40,42–44]. All steps in the B24 platform from sample preparation to data analysis and dissemination are freely accessible to users that have been awarded time through the synchrotron's peer-review process and fully documented via peer-reviewed publications, website protocols and instructional videos [33–35,45,46]. Users can receive training on the use of instruments and software and are expected to collect their own data (remotely or in-person on site) and are highly encouraged to deposit it once published to public archives to allow unfettered access and opportunity for scrutiny to colleagues in the same or related fields [46]. The user base at beamline B24 are predominantly academics investigating areas of interest within the biomedical sciences and who target systems such as: pathogen–host interactions, immunity, cell death, cancer, vaccine and drug development as well as basic cellular biology mechanisms and response to environmental stressors [22,47–55].

## SXT in the aid of biomedical research and the role of correlative approaches

**Figure 4.**
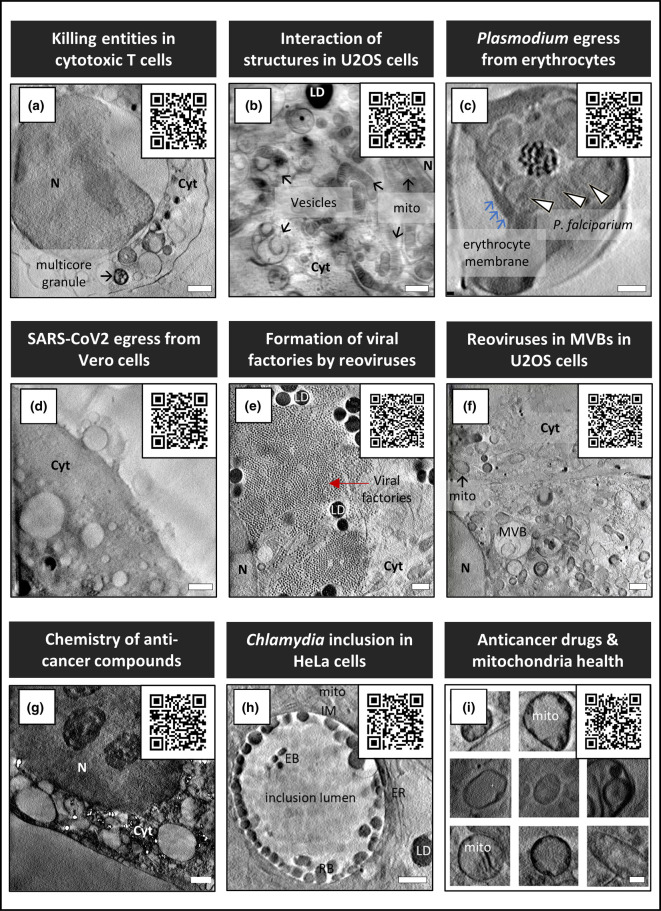
Exemplar studies emanating from the imaging platform at B24. (**a**) Cytotoxic T-cells producing attack particles [50]. (**b**) Human osteosarcoma cells (U2OS cells) showing the localization of filamentous actin with vesicles as well as the location and communications between other microstructures [49]. (**c**) Loss of mechanical integrity of erythrocyte membrane in the final stages of *Plasmodium falciparum* egress [51]. (**d**) SARS-CoV2 egress from Vero cells [47]. (**e**) Tracking of the intracellular pathway of reoviruses shows the formation of viral factories [22]. (**f**) Localization of reoviruses in multivesicular bodies [22]. (**g**) The selective cellular uptake and localization of photo-activatable anti-cancer compounds [48]. (**h**) Distribution trends of *Chlamydiae* populations in intracellular inclusions in infected human cells [53]. (**i**) Changes in mitochondria morphology due to an organoiridium photosensitiser [52]. Scale bars = 1 µm. N = nucleus, Cyt = cytoplasm, LD = lipid droplet, MVBs = multivesicular bodies, ER = endoplasmic reticulum, EB = elementary bodies, IM = inclusion membrane, mito = mitochondria. Images were used with permission.

Representative studies aided by SXT include, the discovery of a new cytotoxic membrane-less organelle that killer T-cells deposit on the surface of their targets (Figure 4a), the tracking of F-actin structures in mammalian cells (Figure 4b), the charting of plasmodium egress mechanisms in infected human red blood cells (Figure 4c), SARS-CoV2 intracellular progress and egress in the late stages of infection (Figure 4d), reovirus vesicle escape in the early stages of infection (Figure 4e,f), the selective cellular uptake and localization of photo-activatable anti-cancer compounds (Figure 4g), distribution trends of *Chlamydiae* populations in intracellular inclusions in infected human cells and (Figure 4h), mitochondrial targeting by an organoiridium photosensitiser (Figure 4i) [22,47,49–53]. Bioactive nanoparticles have been characterized with regards to their internalization and compartmentalization in cells using 3D cryoSXT [56]. The interaction between superparamagnetic iron oxide nanoparticles (SPION) and cancer cells has been quantitatively explored, serving as a basis for metal-based nanoparticle fabrication [56]. Exploration by cryoSXT also lays the foundation for the adoption of suitable image diagnosis, treatment and drug delivery strategies in nanobiomedicine. The ability of cryoSXT to resolve and contrast viral particles, fungi and bacteria in their near-physiological state makes it a cornerstone tool in the formulation of reagents which show exceptional promise in annihilating these pathogens. This stems from the practical experience that cryoSXT captures the cellular landscape while displaying the dynamism of different components of the cell in response to triggers. Pathogen internalization, assembly and clearance or egress have been widely studied using cryoSXT, thus, the next leap towards bettering human, animal and plant health would be to leverage these acquired knowledges for life-changing solutions [19,20,22,47,51]. A lot of discoveries have been made with cryoSXT as the main imaging tool but in recent years the combination of methods alongside SXT have provided an exciting new way of increasing the dimensionality of the data through correlative imaging.

## Cross-talk of cryoSXT with other imaging methods

It is unlikely that an imaging method will yield all desired information needed in a study, thus making correlative imaging a powerful approach bringing together the powers of disparate microscopes to produce augmented imaging that propel innovations and discoveries in the life sciences [57,58]. The ability of a technique to not ‘trap’ samples but allow their transfer to other imaging workflows to gain information otherwise not obtainable from just one setup is desirable. CryoSXT in particular can be correlated with other imaging methods and can be used with a view of cascading samples to other imaging instruments for acquisition of extra data on the same sample. Thus, allaying any concerns with respect to data faithfulness and ability to correlate.

### Visible light and X-ray fluorescence

CryoSXT can be successfully paired with visible light and X-ray fluorescence microscopy [22,33,50,55,59]. SXT with SIM have allowed the tracking of viral particles through human cells and answered the question of intracellular capsid delivery of reovirus particles from transporting host vesicles [22]. Another complementary super-resolution fluorescence technique to cryoSXT which is currently under development at beamline B24, is cryo-dSTORM. However, room temperature dSTORM of fixed cells paired with cryoSXT has been successfully employed in studying the formation of cholesterol crystal structures in culture models of atherosclerosis [60,61].

Taking advantage of the tunable wavelength obtainable at synchrotron facilities and imaging at different element's K/L-edges, X-ray absorption near-edge spectroscopy (XANES) has also been used alongside SXT to observe, map and quantify elements (such as calcium, oxygen and phosphorus) in cells or materials [62,63]. An example is seen in studies that have surveyed the involvement of calcium in biomineralization processes in bone tissues [35,62]. Also, for the first time to our knowledge, a combination of cryoSXT, nanofocused X-ray fluorescence (XRF) and XANES spectroscopy were used to establish the near-native single-cell chemistry of an anti-cancer compound- photoactivatable diazido platinum (IV) complexes [48].

### Spectroscopy

The integration of XANES alongside water window soft X-ray absorption tomography on the same microscope has been implemented at beamline MISTRAL at the ALBA synchrotron [62,64,65]. There, they access the full spectral range to 1.5 KeV and with such an expanded spectral range, more structural visualization is enabled to include elemental compositions (K, P, Ca) and imaging deeper into samples already imaged by SXT. At this same beamline, cryoSXT has been paired with synchrotron-based Fourier transform infrared microspectroscopy (SR-μFTIR), albeit, on different microscopes and different sample preparation methods [66]. Pairing cryoSXT with XANES at non-cryogenic temperatures has also been documented using higher-energy X-rays on samples that have been recovered and freeze-dried after SXT [48].

### Electron microscopy/tomography

CryoSXT has also been paired with cryo-electron tomography (cryoET) [47] and can be paired with cryo focused ion beam milling scanning electron microscopy (cryoFIB-SEM). This workflow will normally start with SXT imaging due to the low radiation dose delivered when compared with EM exposures. For the purposes of SARS-Cov-2 studies, for example, SXT provided robust statistical analyses of a large number of infected cells documenting ultrastructural rearrangements and providing the cellular backdrop for the nanometer resolution-targeted EM study of specific areas of infection [47]. At this juncture, it is important to point out that to minimize radiation damage during SXT, careful dosing of samples with X-rays is the best practice and this can be achieved through lower exposure time and larger angular step data collection.

Finally, studies that will benefit from any technique must be carefully mapped and the entire expected returns should be thought through with experts of each imaging tool, where the advantages and drawbacks are taken into consideration before commencing.

## Deciding to use cryoSXT: choices and consequences in imaging

Prior to the commencement of any biological research project which may require the visualization of native cellular features, there are factors to carefully consider in order to guarantee the best possible outcome and ensure the best use of resources. The basic determinant in adopting an imaging method is the subject matter under investigation. Depending on the requirements for dimensionality, the scale of view, detail content and event resolution, selected imaging methods present themselves as best-suited for individual research projects. CryoSXT has been developed to address specific challenges in biomedical research topics in accordance with such requirements and can be the ideal method to capture native 3D information but only following a careful examination of its relevance and potential.

**Figure 5.**
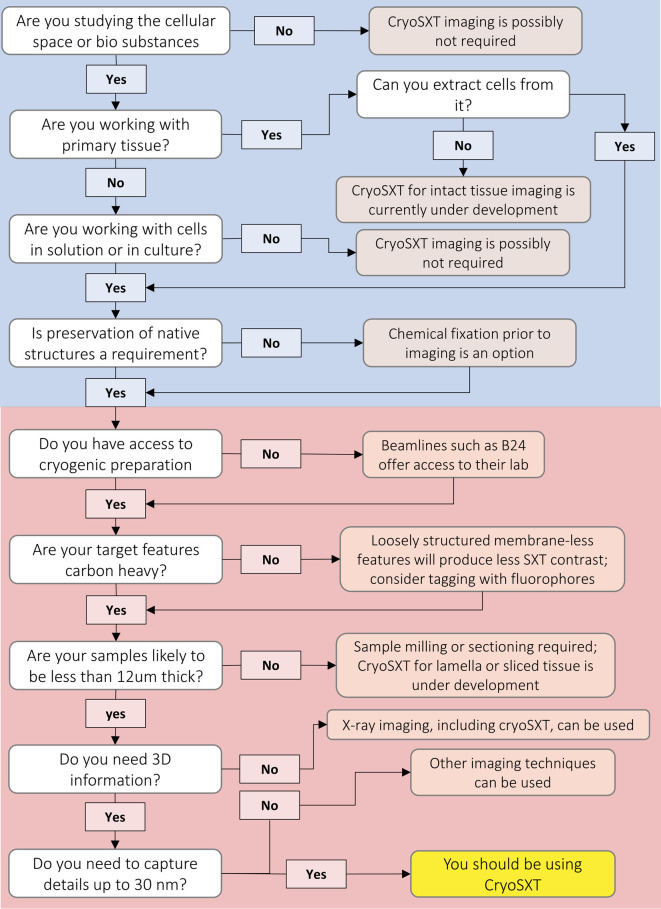
Decision tree leading to cryoSXT use based on project requirements and availability of resources. The section boxed in blue highlights sample/project-specific parameters while the section boxed in red denotes technology-related decisions.

There are a few discrete variables to consider before imaging (Figure 5). In specific:

### Field of view versus resolution

Image resolution gained is normally accompanied by a reduction in the area and volume that can be sampled at that resolution [67–69]. Some microscopy techniques offer generous fields of view (FOVs) with poor resolution while others would trade high resolution for extensive FOVs. Hence, some debate is warranted as to what each project stands to gain before settling on suitable imaging methods to adopt. Higher resolution (≤200 nm) microscopes enable detailed assessment of finer biological features, while lower-resolution setups stand out too due to the increased FOV and depth of focus they deliver (mm or beyond). This makes lower-resolution platforms ideal for studies in need of top-level intercellular tissue-level organization. On the other hand, advanced imaging techniques such as SXT can deliver both highly defined datasets with enhanced FOVs. They provide sufficient resolution to visualize the cellular landscape as it responds to environmental cues, spatially characterize organelles and substructures that drive trafficking and, provide high-content information on structural changes of cellular features in response to specific cues [21,22,70]. SXT, in specific, delivers resolution of features to 25 nm with possible FOVs of tens of micrometers in cells and cell populations up to 10 µm thick in 3D [22,24,29]. This places SXT well within the capacity to deliver clear cellular imaging and, given the sizeable FOVs achieved (anywhere between 10 and 20 μm^3^ in 3D and 70 µm^2^ in 2D), can document cellular ultrastructure through swathes of intracellular and pericellular space in 3D [22]. For a clear understanding of the cellular landscape in 3D, the FOV can be expanded by stitching adjoining areas to make a montage of 3D data [22] that can lead of cumulative FOVs of hundreds of micrometers across. Therefore, with cryoSXT, provided there exists an abundance of carbon-heavy structures such as membranes in cellular components, resolving features within this landscape is attainable.

### Resolution versus contrast

The achievable resolution of a system is not the only vital factor to be considered in microscopy; image contrast, the ability to distinguish between signal and background, underpins the real capacity of a microscope to deliver clear imaging up the promised specifications (Figure 5). Microscopes rely on specialized approaches to differentiate compounds of interest from the background. For instance, fluorescence microscopes rely on fluorescent-emitting chemicals and proteins for signal, bright-field microscopes generate contrast by attenuation of transmitted light in dense areas, while atomic force microscopes read out force and topographic measurements based on a sample's surface features [71–74]. Hence, the contrasting regime should be considered before commencing a project, to ensure the compatibility of chosen visualization schemes with the samples and expected outcomes of the project. This is because, despite the super-resolving power of a given microscope, poor contrast can drown the capabilities of any high-end system while attempts to enhance contrast could lead to serious structural artifacts in the sample. Given that SXT imaging depends on cellular content alone, there is no requirement for contrast-enhancing chemicals to be added and therefore the information captured does not have to be altered in any way by foreign material [18,21,22,29]. However, the native intracellular environment of cells is full of solutes and in SXT this also contributes to an increase in background noise which limits the effectiveness in cell resolution to 30 nm when using objectives that should theoretically perform to better than 25 nm.

### Room temperature versus cryogenic temperature

While some projects will benefit from fixed or dynamic room temperature sample imaging, others require in-depth fine ultrastructural definition and nanometer localization which is unattainable at room temperature without chemical fixation or dehydration. To see cellular structures within their native environment, cryopreservation can be the right approach to both preserve biological information and deliver highly resolved imaging [75,76]. It also negates the requirement for samples to be dehydrated, resin-embedded, fixed with harsh chemicals which cross-links proteins or permeabilized to allow dye uptake. Cryopreservation immobilizes samples ensuring minimal drift during data acquisition and protects them from radiation damage through exposure to the harsh X-ray light that is required to see cell structures in greater detail, an advantage which chemical fixation likewise gives at room temperature [22,28]. For SXT (Figure 5), uniform sample vitrification devoid of crystal ice formation must be optimized to avoid artifacts and ensure consistency and reproducibility [33]. Furthermore, following cryopreservation, these samples must be consistently maintained in a cryogenic state to avoid devitrification and accumulation of crystal ice and therefore the ability of a facility to handle and store vitrified samples will determine the scalability of its operations in this regime [73,77]. Ideally, the humidity within experimental laboratories that rely on cryo-imaging must be maintained much lower than ambient (ideally 20% or less) to avoid ice contamination of samples [22,33].

### User-accessible versus user-friendly

An accessible and well-documented imaging platform is one that can be easily adopted by new users. Nowadays, cryoSXT as a technique has reached a level of maturity at synchrotron settings that makes it accessible to all interested parties (following the peer-review of research proposals) who are willing to follow local health and safety rules and regulations. Once there, the user is typically supported by staff throughout data collection via established user support procedures. Moreover, the method is now extensively documented in instructional publications [33–35,45,46] and dedicated material to reflect onsite specifics (https://www.diamond.ac.uk/Instruments/Biological-Cryo-Imaging/B24.html) which further ensure that the method is accessible to all.

As shown in Table 1, there are only five operational synchrotron-based SXT stations worldwide, therefore, making them naturally oversubscribed. A limited number of proposals are awarded beamtime per period, making access rather limited to a selected group of scientists. This has resulted in SXT not being widely used as it should be. It is hoped that laboratory-based SXT sources would fill this gap in user demand for SXT and reduce the pressure caused by overbooking at synchrotron settings.

### High throughput versus targeted data collection

Emphasis is continually placed on automation and high throughput data collection for microscopy setups across facilities. Automation improves user experience and often reduces stress. There also lies a potential conflict in the volume versus quality of data acquired. High throughput data collection can at times come at a cost when data mining is not automated because data accumulation in turn results in a drain of resources because they need to be assessed and mined. Therefore, a high throughput approach might not always be palatable and can erode the efficiency of data mining and the quality of the data itself. For instance, while one project might benefit from collecting data from features at the cell periphery, another study could benefit from collecting data at the nuclear region; in both cases, the strategy employed to collect data would need to be tailored to that environment and might require one-to-one attention for each single data set collected. Despite the seeming downsides to high throughput data collection, fast multimodal acquisition speeds up and simplifies workflows while also providing unique contextual insights into samples. Thanks to automation, vital research is propelled via remote access and spatial biology applications can be expanded by characterizing an unprecedented number of biomarkers. However, to extract the valuable embedded in multidimensional images, full processing and analysing power is essential and this is not always attainable.

With cryoSXT, both targeted and high throughput data collection strategies are adoptable on a case-by-case basis, influenced by project needs alone. The high throughput nature of cryoSXT is fueled by its fast data collection times of 5–20 min per data set which is overall dependent on data collection strategies that affect the exposure time, angles of rotation and angular step size [22,29].

### Dynamic versus snapshot imaging

Dynamic imaging involves visualizing processes over a period whereas snapshot imaging involves capturing events at specific instances in their entire lifetime. Snapshot imaging is aided by fixation, whether chemical or cryogenic, whereas with live imaging, specimens need not be immobilized, and this can take place in cells, tissues, organs or entire systems. It is almost every investigator's desire to get the best possible maximum resolution at the maximum field of view in a dynamic fashion and in a fully integrated manner. Albeit there are some compromises; does the investigator desire to see events unfold or do they want to trap it in time? Live cell imaging techniques which image deeper and gentler are desirable in the present-day world. CryoSXT offers only snapshot imaging at specific time points as windows into the cellular terrain. Hence, dynamic processes can be captured as they unfold through a series of stills snapped at different time points.

CryoSXT is a unique X-ray 3D imaging technique in that it allows close-to-native imaging of the native unperturbed intracellular world with the minimal amount of sample processing.

## Conclusions

CryoSXT is a tool which is bridging the divide from research to product development and beyond. It's potentials span across cross-disciplinary areas from medicine to environmental monitoring for ecosystem sustainability [54].

## Perspectives

- The field of SXT at cryogenic temperatures fills the need for three-dimensional imaging of biological matter in their near-native state and can enhance our understanding of the cellular landscape and the applicability of biomaterials towards solving real-life problems.
- Currently, methods development center on improved resolution and contrast, diversifying the range of samples that qualify for imaging on this platform and software development and improvements.
- In the future, the goal will be to establish cryoSXT as a go-to technology for high throughput medical diagnosis of biopsies, environmental monitoring and as a screening platform for materials of biomedical importance without restrictions on expected outcomes.

## Abbreviations

cryoET: Cryo-electron tomography
cryoFIB-SEM: cryo focused ion beam milling scanning electron microscopy
cryoSXT: cryogenic temperatures
EM: electron microscopy
FOVs: fields of view
FZP: Fresnel zone plate
SPION: superparamagnetic iron oxide nanoparticles
TEM: transmission electron microscopy
TXM: transmission X-ray microscope
XANES: X-ray absorption near-edge spectroscopy
